# Development and initial psychometric evaluation of a questionnaire for post intensive care recovery - PIR

**DOI:** 10.1186/s41687-026-00993-7

**Published:** 2026-01-18

**Authors:** Anna Eriksson, Lotti Orwelius, Kristofer Årestedt, Michelle S. Chew, Marika Wenemark

**Affiliations:** 1 Department of Anaesthesia and Intensive Care, Linköping University Hospital, Linköping, Sweden; 2 Biomedical and Clinical Sciences, Linköping University, Linköping, Sweden; 3 Faculty of Health and Life Sciences, Linnaeus University, Kalmar, Sweden; 4 Department of Research, Region Kalmar County, Kalmar, Sweden; 5 Medicine and Caring Sciences, Linköping University, Linköping, Sweden; 6 Department of Health, Medicine and Caring Sciences, Faculty of Medicine and Health Sciences, Linköping University, Linköping, Sweden; 7 Department of Women’s and Children’s Health, Karolinska Institutet, Stockholm, Sweden; 8 Department of Health Data Analysis, Region Östergötland, Linköping, Sweden

**Keywords:** Intensive care, Outcome, Rehabilitation, Self report, Patient reported outcomes measures, Questionnaire development, Methodological study, Psychometrics

## Abstract

**Background:**

Recovery after intensive care is a complex and sometimes prolonged process. There is no instrument available for measuring the physical, psychological, and social dimensions of recovery in this setting. The extent of impairment in each dimension and their relative contributions to well-being after survival from critical care is unknown. The aim of this study was to develop a standardised questionnaire for measuring recovery during the whole process after intensive care and evaluate its psychometric properties with focus on factor structure and internal consistency.

**Method:**

The study was performed in two different phases, the development and psychometric evaluation. The development phase included four-steps: (1) identification of possible domains and items of relevance; (2) Delphi study to achieve consensus on critical items to include in the questionnaire; (3) questionnaire construction; (4) cognitive interviews. The psychometric evaluation phase was based on data from 166 patients, recruited from two general ICUs in Sweden. Ordinal confirmatory factor analysis (CFA) was conducted to examine the factor structure, and ordinal alpha and ordinal omega were used to assess internal consistency reliability.

**Results:**

The preliminary version covering the domains of Psychological, Symptoms, Cognition, Daily activity, and Personal resilience in Post Intensive care Recovery (PIR). The hypothesised dimensions were confirmed in the CFA after some theoretically motivated adjustments, and all scales demonstrated satisfactory internal consistency reliability (α = 0.78–0.93, ω = 0.73–0.92).

**Conclusion:**

This study suggests that the PIR demonstrates sound psychometric properties in factor structure and internal consistency. However, further evaluation is required before it can be recommended for clinical use.

**Trial registration:**

Not applicable.

## Background

Advancements in healthcare methods have led to more sophisticated medical treatments of critical care patients, resulting in an increased survival rate for patients with critical illnesses. Survivors after intensive care often experience new or worsened physical, psychological, and cognitive problems [1–3], known as PICS (Post-Intensive Care Syndrome) [1], and a majority of survivors experience one or more PICS-related complaints during the recovery phase after intensive care [3–7]. Consequently, the recovery process is essential for restoring wellbeing following intensive care.

There is no unified definition of recovery, and it vary across diseases [8–10]. Allvin et al. has defined postoperative recovery as “*an energy-requiring process of returning to normality and wholeness as defined by comparative standards*,* achieved by regaining control over physical*,* psychological*,* social*,* and habitual functions*,* which results in returning to preoperative levels of independence/dependence in activities of daily living and an optimum level of psychological well-being*” [11]. In the context of intensive care, “recovery” is often defined as the process through which patients regain their health and return to a stable condition [12, 13]. Recovery can include a reduction in unpleasant physical symptoms, improved emotional well-being, regaining function, and returning to one’s usual activities [14]. Detection and treatment of PICS may be one way of increasing recovery [15]. Providing patients with realistic expectations and allowing them to take responsibility in this active and demanding part of their health journey may be important for the recovery process [16, 17]. Common problems in the recovery process, for the patients cared for in an ICU, include psychological aspects, such as depression, post-traumatic stress disorder, and anxiety [1], symptoms like pain, fatigue [18], and dysphagia [19], cognitive impairments such as problems with memory, concentration, attention and processing ability [1, 20, 21], problems in daily activities such as muscle weakness [18, 22, 23], weakened condition, reduced exercise capacity [23], and lack of personal resilience in areas such as emotions, capacity to adjust, and maintaining a positive focus [24]. To identify persons in need of support during the recovery process, there is a need for a comprehensive measure of these problems.

When measuring recovery, it is common to use generic measures not specifically developed for recovery after intensive care [25]. Only one questionnaire has been identified that specifically measures recovery after intensive care, focusing on the social, existential and spiritual aspects of recovery [26]. In addition, O’Neill et al. developed a questionnaire, that addresses a broad range of support needs during key transitions in the recovery process after ICU admission, including informational, emotional, instrumental, appraisal, and spiritual needs [27]. Therefore, there is a need for a self-reported questionnaire for recovery covering a variety of problems that are common among ICU survivors. As recovery is an ongoing process that begins early after discharge from the Intensive Care Unit (ICU) [3, 6], it should be feasible to use the questionnaire during the whole recovery process, including the period shortly after ICU discharge [6, 28]. Early identification could increase the likelihood of a faster return to optimal health, with potential benefits such as reduced sick leave, decreased need for municipal or primary care support services, and improved self-rated health-related quality of life. Consequently, the aim of this study was to develop a questionnaire for measuring recovery after intensive care, the Post Intensive care Recovery (PIR), and evaluate its psychometric properties with focus on factor structure and internal consistency.

## Method

### Design

This methodological study was conducted in two phases, the development of PIR and an initial psychometric evaluation. The Swedish Ethical Review Authority approved the study (No. 2019–05422, and 2022-01957-02).

### The development of the questionnaire

**Fig. 1:**
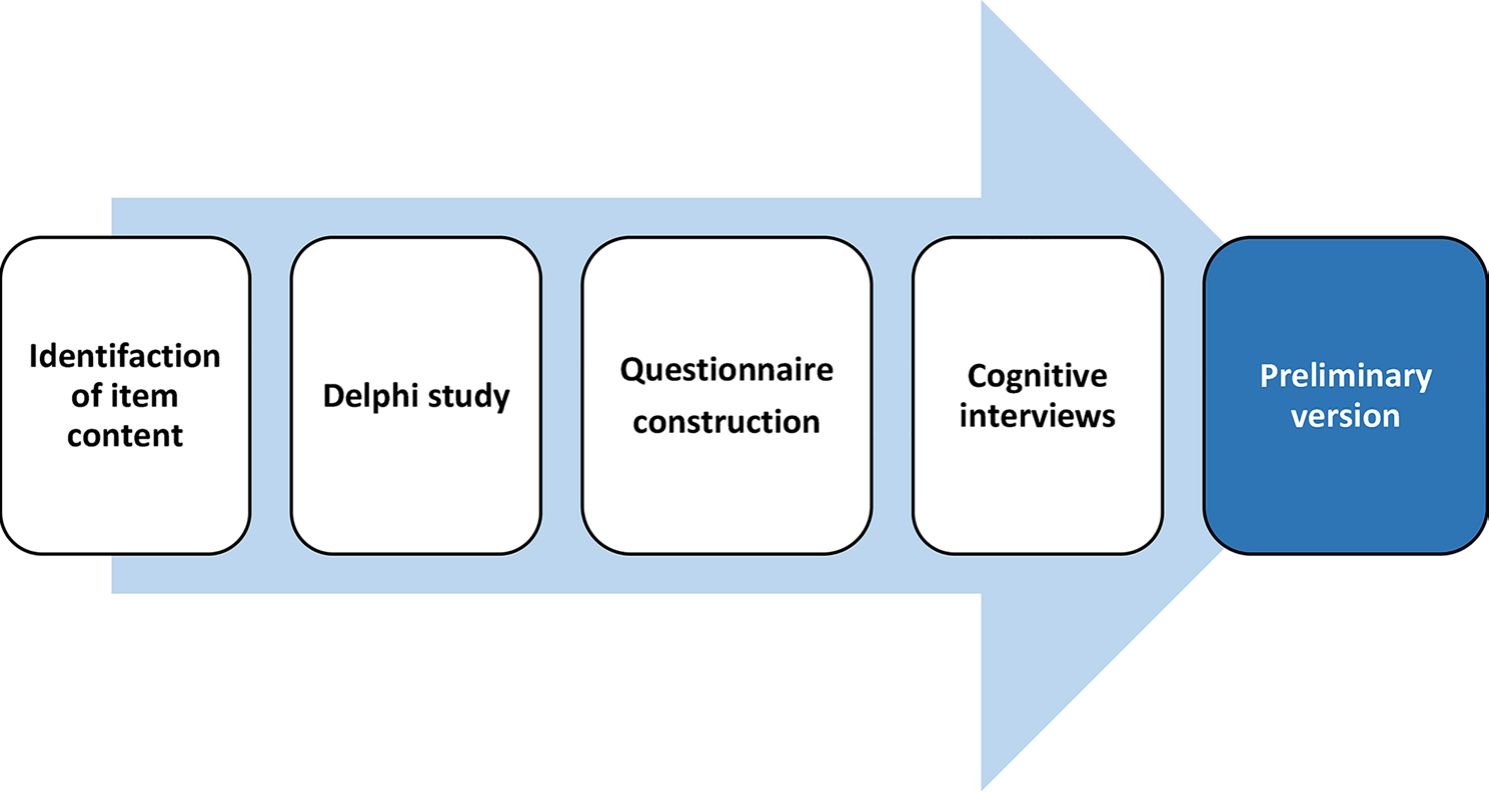
Description of the development of PIR

The development of PIR was inspired by a process described by Heywood et al. [29], that was created to evaluate patient reported outcome measures. In the present study, the development included four steps (Fig. 1).

### Identification of item content

As a first step, the authors (AE, LO, MC) reviewed the literature and identified five questionnaires that measure recovery of relevance to intensive care treatment; Quality of Recovery-40 (QoR-40) [30], Health Related Quality of Life, HRQoL (RAND-36) [31, 32], Hospital Anxiety and Depression Scale (HADS) [33, 34], Post Traumatic Stress Syndrome (PTSS-14) [35], and MFI-20 (Multidimensional Fatigue Inventory) [36, 37]. The questionnaires were reviewed by the authors to identify item content of relevance for early recovery. This initial selection of preliminary items, designed to represent the core aspects of recovery, was guided by knowledge from previous studies, as well as the authors’ clinical experiences from post-ICU care follow up. This process ended up in 47 preliminary items that are all of potential importance for early recovery after ICU and covered five different aspects of recovery that are of central importance in recovery for ICU survivors: psychological, symptoms, cognition, activities in daily life, and personal resilience. A list of these items formed the foundation for the Delphi study.

### Delphi study

In a second step, a Delphi study was conducted to ensure content validity [38, 39]. An email invitation to participate in the Delphi study was sent to members of the Health Service Research & Outcome (HSRO) section at the European Society of Intensive Care Medicine (ESICM) and the European federation of Critical Care Nursing Associations (EfCCNa) (September 2018). In addition, former patients with experiences of ICU care were included.

In round one, the 47 items from step one were included in a web-based questionnaire and the participants were instructed to rate the relevance of each item on a 5-point scale, ranging from *Strongly disagree* to *Strongly agree*. They also had opportunities to comment on each item and suggest additional items [38–40]. The result were analysed with Content Validity Ratio (CVR) using Lawshe’s formula [41]:

$$\:\:CVR=\frac{{n}_{e}-(N/2)}{N/2}$$

In which n_e_ is the number of respondents that answered *Completely agree*, and N is the total number of participants in the expert panel. To determine the lowest level of CVR that can be used to determine if the item has reached consensus or not, the CVR_critical_ table developed by Ayre and Scully was used [42].

The response rate was 87.5% and the Delphi expert group (*n* = 24) consisted of nine physicians, eight nurses, three physiotherapists, and four patients, who were from Australia, Canada, and five countries in Europe. All health care professionals had experience in recovery and outcome measures after intensive care. In the first round, nine out of the 47 items reached a CVR > 0.70, demonstrating a level of consensus high enough for direct inclusion and were therefore not included in round two. The remaining items were sent in a second round to all health care professionals that had answered the first round. They were instructed to rate the importance of each items on a 3-point scale (*Disagree*,* Agree but not essential*,* Agree*) [41], and considering the entire process of recovery as well as importance from the patients’ perspectives. All assessments were made independently, without access to other experts’ responses. The response rate in the second round was 85% (seven physicians, eight nurses, and two physiotherapists). Consensus was reached in 15 of the 38 items (CVR ≥ 0.53). The nine items that reached consensus in the first round, along with the 15 items in the second round, resulted in a total of 24 items.

### Questionnaire construction

As a third step, after the Delphi study, an initial version of the questionnaire was developed. The preliminary items were formulated as questions and grouped into sections with suitable headings. Fundamental principles were that questions should cover both difficulties and abilities, and should use a simple, accessible language. Response scales were designed to reflect how patients would naturally respond to a question, ensuring that patients would easily be able to find a suitable response option. During the construction phase, items were refined to avoid redundancy, and adjustments were made to ensure coherence of the overall questionnaire, resulting in 30 items.

A supplementary list of items that had not reach consensus in the Delphi study was compiled. This list was used in the next development step (cognitive interviews), with purpose to ensure that potentially relevant items, which may have been undervalued by the Delphi expert group were reconsidered from a patient perspective. Patients were asked to evaluate if any items on the supplementary list should be added to the questionnaire. To further ensure content validity from the patients’ perspectives, an open-ended question asking about items they thought were lacking in the questionnaire was added.

### Cognitive interviews

Six cognitive interviews were conducted to examine if items could be understood and answered as intended and to further ensure content validity [43]. Patients were invited to the interview either when contacted by a social worker from ICU to schedule the patient for a post-ICU follow-up visit within two to four months after ICU discharge, or by a designated nurse at the care unit within one week after discharge from ICU. The inclusion criteria were ≥ 18 years old and ICU Length of Stay (ICU LoS) > 48 h. A convenience sampling method was used, which led to a variation in age, sex, and discharge time after ICU. Patients were interviewed at the end of planned post-ICU follow up (*n* = 4) or at a care unit visit (*n* = 2). The interviews were conducted in Swedish by two of the researchers AE (intensive care nurse) and MW (survey methodologist) and lasted between 11 and 22 min.

The interviews started with a “think aloud” phase followed by retrospective probing and were audio recorded and transcribed verbatim. Identified problems were analysed and categorised according to the cognitive response process (understand, retrieve information, estimation, and response) [44], leading to a revision of the questionnaire. A few items were re-worded for clarity, others were re-worded to reduce cognitive difficulties (for example, by not mixing positive and negative questions within the same section). Some items were excluded, and additional items from the supplementary list were added, for example, items regarding personal resilience such as *A positive view of the future* and activities in daily living such as *Physical activities*. One of the response scales that ranged between *None of the time* to *All of the time* was changed to a scale that ranged between *Never* to *Always* since the respondents asked for an options for *Now and then* and had difficult to answer some of the items, for example for symptoms of dizziness. Another example is that the item *Manage your usual activities* was misunderstood by some patients. It was therefore rephrased to *Manage your work or your usual chores* and added to a new section together with items on managing different aspects. When finalising the questionnaire, two additional questions about perceived recovery status today versus estimated recovery status in one year were added to take into account patients’ views on their overall recovery process. Further, three questions about the patients’ physical, psychological, and general health status before the critical illness period were added to control for health status prior to ICU admission.

#### Preliminary version and scoring

The preliminary version consisted of 34 items, reflecting all five domains of recovery: Psychological (five items), Symptoms (six items), Cognition (six items), Daily activities (seven items), and Personal resilience (five items), ending with the added five questions concerning health status before critical illness period and recovery status today versus in one year that are not intended to be part of the measurement scales. *Loss of sensation* that were included during the pandemic were excluded and *Can do nothing about your situation* were redundant with *Frustrated about your situation* and therefore excluded from the psychometric evaluation.

Four different response scales were used for the different items: *Never* to *Always* (0–4), *No difficulties* to *Major difficulties* (0–3), *Managed completely* to *Didn’t manage at all* (0–3), and *Very much* to *Not at all* (0–3). Each scale score is computed and reported as a sum of item scores.

### Initial psychometric evaluation

#### Statistical analyses

Descriptive statistics were used to present the background characteristics of the participants and distribution of item and scale scores. Overall, continuous data were presented with means and standard deviations, ordered categorical data (ordinal) with median (Mdn) and quartiles (Q1, Q3) and non-ordered categorical data (nominal) with frequencies and percentages.

Descriptive statistics for ordinal responses were used to evaluate data quality, i.e., item and scale score distributions and item non-response patterns. Floor and ceiling effects were also evaluated for the scale scores, defined if more than 20% were distributed at the extremes, i.e., highest or lowest possible score [45, 46].

A series of confirmatory factor analyses (CFA) were conducted to evaluate factor structures. To handle the ordinal nature of data, the parameters were estimated using the weighted least squares means and variance adjusted (WLSMV) estimator, which is based on polychoric correlations between items [47]. First, a baseline model for each dimension was examined; this unidimensional model did not include any modifications, except that response categories were collapsed for alternatives that were endorsed by fewer than 10 persons. The model fit was evaluated using goodness of fit indices, parameter estimates (e.g., factor loadings), and examinations of Haywood cases. We used goodness of fit indices that covered absolute fit (standardised root mean square residual, SRMR), parsimony correction (root mean square error of approximation, RMSEA), and comparative fit (Comparative Fit Index, CFI and Tucker-Lewis Index, TLI). A good model fit is characterised of SRMR ≤ 0.08, RMSEA ≤ 0.06, CFI and TLI ≥ 0.95, strong factor loadings, small and uncorrelated error variances, and absence of Heywood cases. As different criteria have been suggested for the fit indices, we interpreted RMSEA ≤ 0.08, TLI ≥ 0.90, and CFI ≥ 0.90 as acceptable fit [47]. The modification index and residual correlation matrix was inspected to identify problems with the hypothesised models. Based on these, theoretically motivated modifications were made if needed.

Ordinal alpha (α) and ordinal omega (ω) were used to evaluate internal consistency reliability [48, 49]. An alpha or omega ≥ 0.7 was considered as satisfactory [50].

Descriptive analyses were conducted in SPSS version 29.0.1.1 (IBM Corp., Armonk, NY, US) and the psychometric analyses in R 4.4.2 (R Foundation for Statistical Computing, Vienna, Austria), including the lavaan 0.6–19, psych 2.4.6.26, and semTools 0.5-6 packages.

### Method

To evaluate the psychometric properties of PIR, data was collected at two general ICUs, a nine-bed unit at a University Hospital and a six-bed unit at a regional hospital, both in the southeast of Sweden. Patients were recruited between May 2022 until November 2023 on five occasions; May 2022 (patients who had ICU care during the last 12 months) and September 2022, January 2023, June 2023, November 2023 (patients under care during the last three months). The inclusion criteria were *≥* 18 years of age, ICU LoS > 48 h, ICU discharge from 14 days up to three months - one year, ability to speak, read, understand Swedish, and a level of consciousness GCS > 10 or RLS < 4. Patients were invited by mail with a cover letter, an informed consent form, the preliminary version of the recovery questionnaire, and a prepaid return envelope. Background data such as age, sex, ICU LoS, and diagnosis were collected from medical records.

## Result

### Study population

Of 318 patients contacted, 166 (52%) answered the questionnaire. The median age was 68 years, and the majority were men (66%). The most common diagnosis group at ICU was pulmonary diseases such as COVID-19, pneumonia/ARDS or respiratory insufficiency (*n* = 67, 40%). More information about the patients is presented in Table 1. Fifteen patients (9%) needed help from someone else to answer the questions.

**Table 1:** Patient characteristics (*n* = 166)

Age (years), Mdn (min-max)	67 (21–90)
Sex, n (%)	
Female	57 (34)
Male	109 (66)
SAPS 3, Mdn (min-max)	57 (33–102)
ICU LoS (hours), Mdn (min-max)	121 (33-2106)
Duration of invasive mechanical ventilation (hours), Mdn (min-max)	65 (0-1862)
Diagnosis at admission (*n* = 165), n (%)	
Pulmonary diseases	66 (40)
Infection	27 (16)
Gastrointestinal	24 (15)
Other	16 (10)
Cardiovascular	12 (7)
Neurological	11 (7)
Trauma	8 (5)

SAPS3 (Simplified Acute Physiology Score), ICU LoS; Intensive Care Unit Length of stay

### Score distribution and item non-response pattern

Overall, all response categories were used, except the worst alternative for the items about *Confusion*,* Speaking*,* and Willingness to fight*. Most of the patients (84%) answered all items, and the frequency of item non-response ranged between 0 and 2.6% (Table 2).

All scales except the Symptom scale and Ordinary life activities in the Daily activity scale demonstrated positive skew distributions. Floor effects were identified for the psychological scale, Cognition scale and Basic activities in the Daily activity scale, while no ceiling effects were detected for any scale. The full range of the scales were only used for the Ordinary life activities in the Daily activity scale (Table 3).

**Table 2:** Item score distribution and missing data patterns

**Do you currently have any of the following?**											
		**Never**	**Rarely**	**Sometime**	**Often**	**Always**	**Missing (n)**				
1	Pain	23.5	24.7	27.2	17.3	7.4	4				
2	Nightmares	56.1	26.2	12.8	3.0	1.8	2				
3	Lack of appetite	43.6	20.9	20.2	12.3	3.1	3				
4	Felt anxious	42.1	22.6	18.9	10.4	6.1	2				
5	Felt depressed	30.9	24.2	30.3	12.1	2.4	1				
6	Had feelings of panic	67.9	15.8	10.3	5.5	0.6	1				
7	Felt confused	58.5	19.5	15.2	6.7	0	2				
8	Felt dizzy	42.7	23.2	26.2	6.1	1.8	2				
9	Felt exhausted	17.0	19.4	35.2	21.8	6.7	1				
10	Loss of sensation	46.1	15.8	12.7	10.9	14.5	1				
**Have you experienced difficulties with the following in the current situation?**											
		**No difficulties**	**Minor difficulties**	**Moderate difficulties**	**Major difficulties**		**Missing (n)**				
11	Breathing	64.6	23.8	11.0	0.6		2				
12	Sleeping	49.4	24.4	15.9	10.4		2				
13	Speaking	82.3	15.2	2.4	0		2				
14	Concentrating	53.9	30.3	13.3	2.4		1				
15	Swallowing	74.1	19.3	4.2	2.4		0				
16	Remembering things	42.8	38	15.1	4.2		0				
**How did you manage the following in the current situation?**											
		**Managed completely**	**Managed mostly**	**Managed to some extent**	**Didn’t manage at all**		**Missing (n)**				
17	Wash yourself and get dressed	78.3	10.8	7.2	3.6		0				
18	Walk 100 m	67.3	10.9	9.1	12.7		1				
19	Using small items like a pen or toothbrush	85.5	9.1	4.8	0.6		1				
20	Communicating with others	84.3	12.0	3.0	0.6		0				
21	Understand instructions and advice	72.7	23.0	3.6	0.6		1				
22	Work or your usual chores	32.7	23.0	24.8	19.4		1				
23	Physical activities (exercising)	22.9	15.7	44	17.5		0				
24	Leisure activities or hobbies	31.1	18.3	26.2	24.4		2				
25	Social relations	53.0	25.9	19.9	1.2		0				
**How often have felt the following in the current situation?**											
		**Never**	**Rarely**	**Sometime**	**Often**	**Always**	**Missing (n)**				
26	Being a nuisance to family and friends	47.9	18.2	23.6	8.5	1.8	1				
27	Can do nothing about situation	34.6	23.3	24.5	12.6	5	3				
28	Frustrated about your situations	24.2	19.4	30.3	19.4	6.7	1				
**Do you feel that you have…**											
		**Very much**	**Quite a lot**	**Not very much**	**Not at all**		**Missing (n)**				
29	…a positive view of the future	39.3	38.7	19	3.1		3				
30	….willingness to fight to get better	71.2	20.9	8	0		3				
31	…confidence that the healthcare system will support you in the future	54.3	27.2	14.2	4.3		4				
**How was your state of health just before intensive care?**											
		**Very good**	**Good**	**Fairly**	**Bad**	**Very bad**	**Missing (n)**				
32	Mental health	45.5	29.7	11.5	9.7	3.6	1				
33	Physical health	23.6	38.2	19.4	9.7	9.1	1				
34	General health	23.6	41.8	23	5.5	6.1	1				
		1	2	3	4	5	6	7	8	9	10
35	How would you say you have recovered from intensive care, at the moment?	1.2	1.8	4.2	10.8	11.4	14.5	17.5	15.7	10.8	12
36	How much do you think you will have recovered from intensive care, one year after intensive care?	0	0	1.8	3	4.8	7.9	6.7	15.2	21.2	39.4

### Factor structure and internal consistency

#### Psychological scale

The Psychological scale included items about *Nightmares*,* Felt anxious*,* Felt depressed Feelings of panic*,* and Sleeping.* The baseline one-factor model demonstrated satisfactory fit according to CFI (0.999), TLI (0.998), and SRMR (0.033), while RMSEA (0.062) demonstrated acceptable fit (Table 4). The factor loadings ranged between 0.71 and 0.96 (Table 5) and no Heywood cases were identified. The scale demonstrated good internal consistency according to ordinal alpha (0.92) and ordinal omega (0.89) (Table 3).

#### Symptom scale

The Symptom scale included items about *Pain*,* Lack of appetite*,* Felt dizzy*,* Felt exhausted*,* Breathing*,* and Swallowing.* The baseline one-factor model demonstrated good fit according to CFI (0.965) and SRMR (0.072), acceptable fit regarding TLI (0.942), and poor fit regarding RMSEA (0.081) (Table 4). An inspection of the modification indices and residual correlation matrix did not identify any clear problems, such as correlated error variances. Consequently, no revised version was examined. The factor loadings for the baseline one-factor model range between 0.47 and 0.77 (Table 5) and no Heywood cases were identified. The scale demonstrated acceptable internal consistency according to ordinal alpha (0.78) and ordinal omega (0.73) (Table 3).

#### Cognition scale

The Cognition scale included items about *Felt confused*,* Speaking*,* Concentration*,* Remembering things*,* Communication with others*,* and Understand instructions and advice.* The baseline one-factor model demonstrated good fit regarding CFI (0.993), TLI (0.988), and SRMR (0.071), while RMSEA (0.072) demonstrated acceptable fit (Table 4). An inspection of the modification indices and residual correlation matrix identified a possible error variance correlation between the items about *Communications with others* and *Understand instructions and advice.* These error variances were therefore allowed to correlate in a modified one-factor model, which showed good model fit according to all fit indices; RMSEA 0.041, CFI 0.998, TLI 0.996 and SRMR 0.052 (Table 4). The factor loadings in the modified one-factor model ranged between 0.73 and 0.90 (Table 5) and no Heywood cases were identified. The scale demonstrated good internal consistency according to ordinal alpha (0.93) and ordinal omega (0.89) (Table 3).

**Table 3:** Scale score distributions and internal consistency reliability

	Possible score range, min-max	Score range, min-max	Median (Q1,Q3)	Floor/ Ceiling effects, %	Skewness	Ordinal Alpha	Traditional Cronbach´s alpha	Composite reliability
Psychological scale	0–19	0–17	4 (1, 7)	20.4 / -	0.845	0.92	0.89	0.92
Symptom scale	0–22	0–16	6 (3, 9)	5.1 / -	0.406	0.78	0.70	0.73
Cognition scale	0–19	0–15	2 (0, 5)	32.1 / -	1.352	0.93	0.83	0.89
Daily activity scale								
Basic activities	0–9	0–8	0 (0, 2)	64.0 / -	1.625	0.93	0.75	0.87
Ordinary life activities	0–12	0–12	5 (2, 8)	17.2 / 0.6	0.015	0.90	0.87	0.89
Personal resilience scale	0–17	0–16	4 (1, 7)	13.0 / -	0.576	0.86	0.79	0.74

#### Daily activity scale

The daily activity scale included items about: *Wash yourself and get dressed, Walk 100 m, Using small things like a pen or toothbrush, Work or your usual chores, Physical activities, Leisure activities or hobbies, and Social relations*. The baseline model demonstrated good fit regarding CFI (0.986) and TLI (0.979), but poor fit regarding RMSEA (0.131) and SRMR (0.084) (Table 4). An inspection of the modification indices and residual correlation matrix identified several problems with correlated error variances for several items. This problem belonged to two different types of items, i.e., basic activities *(Wash yourself and get dressed, Walk 100 m, Using small things like a pen or toothbrush)* and ordinary life activities *(Work or your usual chores, Physical activities, Leisure activities or hobbies, and Social relations)*. Therefore, a two-factor model was considered. This model demonstrated good fit according to CFI (0.997), TLI (0.995), and SRMR (0.054), while RMSEA (0.066) demonstrated acceptable fit (Table 4). The factor loadings ranged between 0.88 and 0.91 for Basic activities and between 0.61 and 0.96 for Ordinary life activities. The factor correlation was 0.82 (Table 5). In addition, no Heywood cases were identified. Both subscales demonstrated good internal consistency according to ordinal alpha (0.93/0.90) and ordinal omega (0.87/0.89) (Table 3).

#### Personal resilience

The personal resilience scale included items about, *Being a nuisance to family and friends, Frustrated about your situation, …a positive view of the future, …willingness to fight to get better and …confidence that the healthcare system will support you in the future*. The baseline model demonstrated good fit according to CFI (0.973) and SRMR (0.071), acceptable fit according to TLI (0.947), and poor fit according to RMSEA (0.150) (Table 4). An inspection of the modification indices and residual correlation matrix identified a possible error variance correlation for the items about *Being a nuisance to family and friends* and *Frustrated about your situation*. These error variances were allowed to correlate in the modified one-factor model, which showed good fit according to all fit indices; RMSEA 0.049, CFI 0.998, TLI 0.994 and SRMR 0.034 (Table 4). The factor loadings for the modified one-factor model ranged between 0.64 and 0.94 (Table 5) and no Heywood cases were identified. The scale demonstrated good internal consistency according to ordinal alpha (0.86) and ordinal omega (0.74) (Table 3).

**Table 4:** Goodness-of-fit indices

	χ^2^ goodness-of-fit		RMSEA					
	χ^2^ (df)	*p*-value	RMSEA	90% *CI*	*p-*value	CFI	TLI	SRMR
**Psychological scale**								
Baseline one-factor model	8.11 (5)	0.150	0.062	0.000-0.137	0.331	0.999	0.998	0.033
**Symptoms scale**								
Baseline one-factor model	18.25 (9)	0.032	0.081	0.023–0.135	0.151	0.965	0.942	0.072
**Cognition scale**								
Baseline one-factor model	16.54 (9)	0.058	0.072	0.000-0.126	0.225	0.993	0.988	0.071
Modified one-factor model*^1^	10.20 (8)	0.251	0.041	0.000-0.107	0.516	0.998	0.996	0.052
**Daily activity scale**								
Baseline one-factor model	52.32 (14)	< 0.001	0.131	0.094–0.170	< 0.001	0.986	0.979	0.084
Modified two-factor model*^2^	22.10 (13)	0.054	0.066	0.000-0.112	0.257	0.997	0.995	0.054
**Personal resilience scale**								
Baseline 1 factor model	23.03 (5)	< 0.001	0.150	0.092–0.215	0.004	0.973	0.947	0.071
Modified 1 factor model*^3^	5.53 (4)	0.237	0.049	0.000-0.137	0.420	0.998	0.994	0.034

*1 correlated error variances between item 20 and 21

*2 a two-factor model without any further modifications

*3 correlated error variances between 26 and 28

**Table 5:** Standardized factor loadings and error variances

Items	Factor loadings	Error variances
**Psychological scale**		
Q2 Nightmares	0.76	0.43
Q4 Felt anxious/ anxiety	0.96	0.09
Q5 Felt depressed/depression	0.92	0.15
Q6 Had feelings of panic	0.93	0.13
Q12 Sleeping	0.71	0.50
**Symptom scale**		
Q1 Pain	0.47	0.78
Q3 Lack of appetite	0.61	0.63
Q8 Felt dizzy	0.62	0.62
Q9 Felt exhausted /Fatigue	0.77	0.41
Q11 Breathing	0.60	0.64
Q15 Swallowing	0.65	0.58
**Cognition scale**		
Q7 Felt confused	0.90	0.19
Q13 Speaking	0.76	0.42
Q14 Concentrating	0.90	0.20
Q16 Remembering things	0.87	0.25
Q20 Communicating with others	0.81	0.34
Q21 Understand instructions and advice	0.73	0.47
**Daily activity scale**		
** Basic activities** ^*^		
Q17 Wash yourself and get dressed	0.91	0.18
Q18 Walk 100 m	0.95	0.10
Q19 Using small items like a pen or toothbrush	0.88	0.23
** Ordinary life activities** ^*^		
Q22 Work or your usual chores	0.92	0.16
Q23 Physical activities (exercising)	0.89	0.22
Q24 Leisure activities or hobbies	0.96	0.08
Q25 Social relations	0.61	0.63
**Personal resilience scale**		
Q26 Being a nuisance to family and friends	0.64	0.59
Q28 Frustrated about your situations	0.72	0.48
Q29 …a positive view of the future	0.94	0.12
Q30 …willingness to fight to get better	0.68	0.54
Q31…confidence that the healthcare system will support you in the future	0.67	0.56

* Covariance Basic activities-Ordinary life

## Discussion

This study describes the development and initial evaluation of the post intensive care recovery. To our knowledge, this is the first questionnaire to measure recovery during the whole recovery process starting so early after discharge from ICU, covering a wide range of different aspects of recovery. Overall, content validity was supported by experts and patients, the suggested latent structure was confirmed, and the scales demonstrated good internal consistency.

### Development of the questionnaire

In order to develop a comprehensive questionnaire, content validity from both patients’ and health care professionals’ perspective was ensured through several steps: review of other questionnaires, Delphi rounds, and cognitive interviews – a methodological combination that strengthens the development process by integrating the perspective of both healthcare professionals and patients. The Delphi methods was selected for its characteristics to independently get the opinions of many experts without having influenced each other [38, 39]. However, a disadvantage of the Delphi method as used in this study, is that the experts assess each item independently, without the opportunity to consider how the items function collectively within the overall structure of the questionnaire [51]. The cognitive interviews addressed this by assessing the questionnaire as a whole, including the supplementary list with items that did not reach consensus among the Delphi expert group. A few items from the supplementary list that several patients rated as important were added even if they did not meet the Delphi criteria for inclusion. Consequently, the preliminary questionnaire integrates perspectives from both experts and patients.

Many questionnaires focus one-sidedly on problems and difficulties, which can cause patients to see their situation in a negative way. Therefore, with a balance between items regarding abilities and difficulties enables to foster a more positive outlook for the patients when answering the questionnaire and the respondents can focus on what they are able to do instead of just focusing on problems in their situation. This can also have significant implications for healthcare by enabling the demonstration of the patient’s current capabilities, the potential for recovery over time based on their ability to manage various tasks, and the reduction of certain problems.

The patient’s physical, psychological, and general health status before intensive care may influence recovery prerequisites and could be important for healthcare professionals to consider in evaluations of the recovery as well as in planning for future support or interventions. Therefore, three questions regarding self-reported health status before ICU admission are included in a part of the questionnaire. Retrospective assessment of self-estimated health provides valuable insight into the patient´s general, physical and physiological health status before ICU admission.

The development process has been a dynamic process and because of the pandemic, there were periods with less activity [52]. The long development period has nevertheless enabled several opportunities to discuss the process and adapt the questions.

### Initial psychometric evaluation

Item non-response was low for all items, indicating that the items are easy to answer and relevant to the study population. The advantage of having few missing values is that it eliminates the need to drop or impute data, which can introduce measurement uncertainty, which may affect the analysis and interpretation of results [45].

All response options were used, except for three items and for the two questions about overall recovery. This indicates that the response scale is working as intended overall. As the score distribution is dependent on the sample, it seems therefore acceptable to retain the same response options throughout the questionnaire until more evidence is accumulated in future studies.

Overall, the hypothesised latent structure of the five scales was supported in this initial evaluation of the PIR, even if some modifications of the measurement models were needed. The Psychological and Cognition scale demonstrated good or acceptable fit in all indices in the baseline models. In both cases, CFI, TLI and SRMR demonstrated good fit while RMSEA demonstrated acceptable fit. Both scales demonstrated also strong factor loadings, indicating that the items reflect the underlying construct. For the Psychological scale, no clear reason could be found to explain why it just reached a acceptable and not good model fit. In contrast, the Cognition scale reached good model fit also in RMSEA after the residual variances between *Communicating with others* and *Understand instructions and advice* were allowed to correlate. Common reasons for correlated residuals are item overlap, multidimensionality, or methodological aspects, for example reversed scored items [53]. For the Cognition scale, none of these problems were considered to explain this error variance correlation. Thus, this modification was only driven by the modification indices, to understand which impact this had on the model fit.

Problems with correlated error variances were also detected in the Daily activity scale and Personal resilience scale. In the Personal resilience scale, this problem was only addressed to two items about *Being a nuisance to family and friends* and *Frustrated about your situation.* This modification was allowed by methodological reasons since these two items share the same response category scale that differ from the other tree items in this scale. This modification ended up in a model with good fit in all indices.

For the Daily activity scale, the problems with correlated error variances trigged a theoretical discussion within the author group that ended up in a division of the scale into two subscales that could be justified conceptually, Basic activities and Ordinary life activities. This modification was supported by the two-factor CFA, which showed good model fit in all indices, except for the RMSEA, that was above but still very close to the suggested level of 0.06. The two latent factors were strongly, but not perfectly correlated, which indicates that the two suggested subscales are closely related but capture two different aspects of recovery in daily activities. When using it, the subscales should be used separately.

Despite that RMSEA did not support acceptable fit for the symptom scale, no specification problems could be identified from the modification indices or the residual correlation matrix. One possible explanation would be that this scale was evaluated using a reflective CFA model, i.e., that the latent construct is assumed to cause the observed indicators (items). However, this type of model has been criticised in certain situations so the observed indicators are assumed to cause the latent construct. In this case, formative models are sometimes suggested [47]. This is partly suggested by the findings from the present study, as this scale overall had the lowest factor loading. Therefore, the Symptom scale needs to be further evaluated in future studies.

To sum up, after some modifications all scales demonstrated satisfactory model fit, acceptable strong factor loadings. Therefore, the scales can be treated as unidimensional measures. Users of the PIR should be aware that the Cognitive scale and Personal resilience scale had minor problems with correlated error variances. One practical consequence of correlated residuals is that these can have a negative impact on the reliability [47]. Despite this, both scales show satisfactory reliability regarding both ordinal alpha and omega.

### Strengths and limitations

The use of multiple steps, including literature review, Delphi study, questionnaire construction, and cognitive interviews, ensures that the PIR is grounded in both expert and patient perspectives. Patients were involved to ensure that the questionnaire should be relevant and accessible to the target population. This enhances its content validity from different perspective.

This study employed confirmatory factor analysis (CFA) and reliability metrics suitable for ordinal data, supporting the validity and internal consistency of the PIR scales. Although the PIR was originally designed as a multidimensional instrument, each scale was evaluated as a separate unidimensional construct due to the limited sample size in the present study. This constitutes an important limitation, as it prevented examination of factor correlations and identification of potential issues such as cross-loadings and correlated error variances across items from different scales. While the scales can be used as separate measures, future research should evaluate the latent structure of the full measurement model, including all scales. Furthermore, classical test theory introduces sample dependency, which may affect the robustness of the results. Future evaluations using item response theory approaches, such as the Rasch model, are therefore recommended [45].

Due to the pandemic, data collection was restricted to two ICU units, resulting in a somewhat limited sample size. We acknowledge that small samples increase the risk of unstable factor loadings and potential overfitting [47]. However, simulation studies indicate that approximately 150 observations are sufficient for a medium-size CFA model using the WLSMV estimation method [53]. Nevertheless, the small sample size and restrictions to two ICUs may limit the generalizability of the findings. Larger and more diverse samples are therefore needed to evaluate the PIR across different populations and settings.

Although some issues were identified—such as correlated error variances in the Cognition and Personal resilience scales, as well as concerns regarding the latent structure of the Daily activity scale—no revisions to the PIR were made at this stage, as additional evidence is required before any definitive conclusions can be drawn to justify further revisions.

The PIR has not yet been evaluated for its ability to measure recovery over time, which is critical for its intended use in assessing changes at both individual and group level. Therefore, future longitudinal studies are essential to establish measurement invariance across time points and to accurately detect changes in recovery.

## Conclusion

The PIR is the first standardised measurement of recovery in patients during the period after intensive care and addresses five key domains of importance for recovery, making it a valuable questionnaire for post ICU follow-up care. The initial evaluation suggests that PIR overall has sound psychometric properties regarding factor structure and internal consistency. Before it can be recommended for clinical and research use, it needs to be further evaluated in larger and more diverse samples, using analytic approaches that are less sample dependent, such as the Rasch model, guiding further revisions. Additionally, longitudinal studies are of importance to ensure measurement invariance across time points and to detect changes. Given these conditions, the PIR questionnaire may facilitate early identification of the key areas in the ICU recovery process, thereby supporting and guide healthcare staff in determining appropriate interventions and measures.

## Data Availability

The dataset used and analysed during this study are available from the corresponding author on reasonable request.

## Abbreviations

PICS: Post Intensive care symptom
HRQoL: Health related quality of Life
ICU: Intensive Care Unit
PIR: Post Intensive care Recovery
PTSD: Post Traumatic Stress Disorder
HSRO: Health Service Research & Outcome
ESICM: European Society of Intensive Care Medicine
EfCCNA: European federation of critical care nursing Association
CVR: Content Validity Ratio
LoS: Length of stay
RLS: Reaction Level stay: GCS: Glascow Coma Stay
ARDS: Acute respiratory distress syndrome
